# Inferential abilities based on pictorial stimuli in patients with right hemisphere damage: influence of schooling

**DOI:** 10.1590/S1980-57642014DN83000008

**Published:** 2014

**Authors:** Ariella Fornachari Ribeiro, Marcia Radanovic

**Affiliations:** 1 Speech Pathologist, PhD, Department of Neurology, University of São Paulo School of Medicine (FMUSP), SP, Brazil.; 2 MD, MSc, PhD, Department of Neurology, FMUSP, SP, Brazil.

**Keywords:** visual inferences, schooling, right hemisphere damage

## Abstract

Inferences are mental representations, formed through the interaction between explicit linguistic information and an individual's world knowledge. It is well known that individuals with brain damage in the right hemisphere (RH) often fail on this task and that schooling may be a variable affecting this.

**Objective:**

To compare the effect of schooling on an inference comprehension task based on pictorial stimuli in patients with RH lesion.

**Methods:**

The inferential abilities of 75 controls and 50 patients with RH lesion were assessed through the pictorial stimuli from the instrument "300 exercises of comprehension of logical and pragmatic inferences and causal chains". Both groups were stratified into two subgroups according to schooling level: 4 to 8 years and 9 or more years.

**Results and Conclusion:**

Highly educated subjects performed better than low educated individuals, both on intergroup and intragroup comparisons (p<0.0001) for logical and pragmatic inference ability.

## Abstract (pt)

Inferências são representações mentais, formadas através da interação entre a informação linguística explícita e o conhecimento de mundo de um indivíduo. Sabe-se que indivíduos com lesão cerebral em hemisfério direito (HD) frequentemente falham nesse tipo de tarefa e que, a escolaridade pode ser uma variável que afeta tal competência.

**Objetivo:**

Comparar o efeito da escolaridade em um teste de compreensão de inferência a partir de estímulos visuais, em pacientes com lesão de HD.

**Métodos:**

As habilidades inferenciais de 75 controles e 50 pacientes com lesão de HD foram avaliadas a partir de estímulos pictóricos do intrumento "300 exercícios de compreensão de inferências lógicas e pragmáticas e cadeias causais". Ambos os grupos foram divididos em dois subgrupos de acordo com o nível de escolaridade: 4 a 8 anos e 9 anos ou mais.

**Resultados e Conclusão:**

O grupo mais escolarizado apresentou melhor desempenho do que os menos escolarizados, seja nas comparações intragrupos e intergrupos (p<0.0001), tanto na compreensão de inferências lógicas quanto pragmáticas.

## INTRODUCTION

A message conveyed through images stands out from a textual message because it is a form of universal linguistic information of immediate understanding, comprehensible by people with different languages and cultures.^1^ Processing of these images requires the use of linguistic inferences, a component of language, defined as mental representations that allow the construction of new knowledge from data previously held in the interlocutor's memory which is activated and applied to the explicit linguistic information conveyed by a message.^2,3^

Picture description tasks evaluate how fast and accurately the subject can interpret extralinguistic and contextual information.^4^ In addition, the comprehension of inferences facilitates the construction and comprehension of discourse, helps to bridge information gaps, and integrates representations that render greater continuity and consistency to arguments of speech.^5^

Although some reports implicate both left (LH) and right hemisphere (RH) in the inferential process, there is a consensus in the current literature that the RH is predominantly responsible for this ability, particularly with reference to pictorial stimuli.^6,7^

RH damage is described as leading to failures in integrating the elements of a story, impairment in the ability to reject incoherent interpretations of a text, and also deficits in interpreting implicit information. Patients with RH damage often fail to grasp the core idea (gist) of a discourse and encounter difficulties proposing a title for a story or choosing a statement that best summarizes the main theme.^8^ All these skills are important for proper communication. Moreover, the ability to understand messages conveyed by pictorial stimuli is pivotal in our society, as much as the comprehension of auditory (linguistic) stimuli.

For inferential processing to take place properly, activation of large-scale cognitive networks is required, including areas related to language, memory, attention and visual processing.^4,9^ Mental models involve a set of propositions that combine implicit and explicit stimulus information, which are culturally determined and learned from our experience in society.^10^

To the best of our knowledge, two studies have reported the ability of RH-damaged patients to generate inferences from visual stimuli.^11,12^ In the first study,^11^ the authors evaluated the ability of RH-damaged subjects to generate inferences from visual stimuli, controlling for the complexity of the stimuli and educational level. They found that patients with higher educational level performed better than those with lower schooling; also, those pictures with greater visual complexity (i.e., more visual elements in the scene) and that were more complex (i.e., with a higher number of associations) presented more difficulty. Interestingly, inferential complexity had a greater impact on the performance of most participants than visual complexity, both for controls and RH-damaged patients. The second study^12^ compared the inferential abilities of healthy and RH-damaged individuals using pictorial stimuli. The authors found that RH-damaged patients performed poorer than controls on comprehending logical and pragmatic visual inferences, independently of lesion site.

It is well known that schooling interferes directly in tasks requiring cognitive processing.^13,14^ Hamel and Joanette^15^ also evaluated subjects' performance for the comprehension of inferences and their findings showed that schooling influenced the comprehension of inferences in normal subjects and patients with RH damage. However, they used auditory stimuli as the assessment instrument. In Brazil, Ribeiro et al.^16^ investigated the performance of normal elderly on tasks of visual inference using pictures of different degrees of visual complexity and compared the performance of subjects according to schooling level. The results indicated that highly educated subjects maintained the same effectiveness in making inferences, independently of picture complexity, but visual complexity interfered with the low and medium educated subjects' ability to make inferences. The authors suggested a *continuum* in inferential processing, which is directly related to schooling level.

Images are widely used to communicate instructions, warnings, stories, and are embedded in all types of media, from newspapers to movies. Moreover, complex pictures are often used in cognitive tests and rehabilitation instruments, hence the importance of understanding the effect of schooling on brain-damaged subjects' ability to comprehend inferences from visual stimuli.

The aim of this study was to evaluate the performance of RH-damaged patients on a task involving the comprehension of inferences derived from pictorial stimulus according to educational level.

## METHODS

**Participants.** Our sample comprised 75 healthy controls and 50 RH-damaged patients. All participants enrolled in this study met the following inclusion criteria: age older than 18 years, right manual dominance as determined by the Edinburgh Inventory,^17^ and a minimum of four years of formal education.

The RH-damaged group was selected from among stroke patients admitted to the Emergency Room at a tertiary hospital linked to a School of Medicine during a one-year period, according to the inclusion and exclusion criteria. The control group was composed of individuals drawn from the community, recruited at a recreation center located in the same city as the patients.

The exclusion criteria for both groups were: history or evidence of neurologic diseases that could affect perceptual or cognitive functions (except for single stroke in the RH-damaged group), history or evidence of psychiatric diseases; chronic use of alcohol or illicit drugs; use of medications at doses that could impair cognitive performance; non-correctable impairment in auditory or visual function.

Patients in the RH-damaged group had to present a single cortical vascular lesion in the right cerebral hemisphere, confirmed by neuroimaging (cranial computed tomography- CT and/or magnetic resonance imaging-MRI).

To be eligible for study enrollment, participants had to achieve normal scores on the following screening tests: the Mini-Mental State Examination (MMSE),^18,19^ the Semantic Verbal Fluency Test (VFT) in the animal category^20^ and the Clock Drawing Test (CDT).^21^

In order to exclude significant attentional or visuoperceptual alterations frequently present in RH lesions (such as hemianopia and neglect), all volunteers were submitted to the Cancellation Test.^22^ Additionally, the Boston Naming Test (BNT) – compact version (15 items) from the CERAD battery^23,24^ was employed to verify both the individuals' naming abilities (lexicosemantic processing) and basic visual abilities (perception of black-and-white patterns, gestalt) considered necessary to accomplish the visual-based inferential task. Participants had to achieve normal scores in both tests. Depressive symptoms were assessed using the Hamilton Depression Rating Scale - 21 items (HDRS – 21),^25^ and individuals with a score higher than 7 were excluded.

Both groups were stratified into two subgroups according to schooling level:

RH-damaged group - consisted of 29 individuals with schooling of four (4) to eight (8) years and 21 individuals with schooling of nine (9) years or more;

Control group: consisted of 29 individuals with schooling of four (4) to eight (8) years and 46 individuals with schooling of nine (9) years or more;

All participants or their legal representatives signed the consent form prior to enrollment on the study, which was previously approved by the Research Ethics Committee of the institution where it was conducted.

**Instrument.** The ability to perform visual inferences was assessed using the instrument *"300 exercises de compréhension d'inferences logique et pragmatique et de chaînes causales"* (300 exercises of comprehension of logical and pragmatic inferences and causal chains),^26^ which was originally conceived for language rehabilitation and stimulation.

For this study, only the pictorial stimuli were employed, consisting of 13 pictures whose color pattern (black and white), tracing and texture are homogeneous. The participants had to observe a picture (e.g., a scene in which a boy approaches his mother crying with a toothache), and then decide which was the best outcome for the story by choosing from among the alternatives also presented as pictures (in this example, the correct answer corresponds to the scene that show the mother taking the boy to the dentist). The pictures required two types of inferential processing, as follows:

[A] Comprehension of logical inferences: to find the logical cause or consequence of a given situation;
[B] Comprehension of pragmatic inferences: to determine which situation, among the alternatives, is most likely to occur.

This instrument has been used previously in a Brazilian population by Ribeiro et al. (2014),^12^ and was considered adequate for assessing the comprehension of visual inferences.

**Testing and scoring procedures.** Patient evaluations were carried out at least one month after stroke. The average time post-stroke was 16.4 months (SD=19.3).

Participants were instructed to carefully examine the pictures and describe what was happening in each of them, subsequently choosing the best option (also a picture) to complete the previous scene. The stimuli allowed for only one correct answer and no time limit was imposed for performing the task. All tests, including cognitive screening, were administered by the main author during a single 45-minute session, in a silent room.

Scoring was performed as follows: 0 - failed inference comprehension; 1- correct inference comprehension. The maximum score was 6 for logical inferences; 7 for pragmatic inferences; and 13 for total (logical and pragmatic) inferences.

**Statistical analysis.** The control and RH-damaged patient groups were compared for mean values of the demographical variables, and for performances on screening tests and the inference comprehension test. Comparison between controls and RH-damaged patients was carried out using Student's t-test. The equivalence in gender distribution was assessed using Pearson's Chi-square test.

The control and RH-damaged subgroups were then classified according to formal educational level: 4-8 years and 9 or more years. The performance of the four subgroups on the comprehension of inferences was compared using one-way analysis of variance (ANOVA) with Bonferroni post-test for multiple comparisons. The possible effect of age on subjects' performance was tested using analysis of covariance (ANCOVA).

All analyses were performed using the statistical software SPSS^®^ for Windows version 20.0, and a significance level (p) of 0.05 was adopted.

## RESULTS

Table 1 shows the demographic and clinical data of the sample. Both groups were equivalent for the variables age, gender and educational level. Regarding the screening tests, the RH-damaged group showed significantly poorer performance across all tasks compared to the control group. Both groups were euthymic at the time of evaluation.

**Table 1:** Demographic and clinical data of the sample.

Variable		Controls (N=75)			Patients (N=50)		p-value
		Mean (SD)	Range		Mean (SD)	Range	
Age		60.3 (8.5)	39-73		58.1 (12)	29-80	0.2618
Educational Level		9.6 (4.2)	4-16		8.9 (3.2)	4-15	0.33
Sex	M	35			25		0.8550
	F	40			25		
MMSE		28.1 (1.6)	24-30		27 (1.9)	24-30	0.0008
VF Animals		18.3 (3.3)	14-28		15 (2.3)	12-20	< 0.0001
BNT		14.3 (0.4)	12-15		13.5 (1.2)	12-15	0.0001
CT		19.7 (0.7)	17-20		18.3 (1.2)	16-20	< 0.0001
CDT		9.7 (0.6)	8-10		8.6 (0.8)	8-10	< 0.0001
HDRS- 21		0.4 (0.9)	0-3		3.4 (2)	0-7	< 0.0001
Time from lesion (months)		NA			16.4 (19.3)	5-84	NA

SD: standard deviation; MMSE: Mini-mental state examination; VF: verbal fluency; BNT: Boston Naming Test; CT: cancellation test; CDT: Clock Drawing Test; HDRS-21: Hamilton Depression Rating Scale-21 items; NA: not applicable.

The sample comprised 40 women (53.4%) in the control group and 25 (50%) in the RH-damaged group. The mean age and schooling for controls were: 65.4±6.5and 4.8±1.3 years (low educated); 57.1±8.2and 12.6±1.9 years (high educated); the mean age and schooling for RH patients were 59.2±12.2and 6.6±1.7 years (low educated); 56.7±11.8 and 12.2±1.6 years (high educated). Regarding age, low educated controls were older than both high educated controls (p=0.003) and high educated RH patients (p=0.012). ANCOVA disclosed no effect of age on subjects' performance for logical (p=0.138), pragmatic (p=0.094) or total scores (p=0.078) in inference comprehension. Educational level exerted an effect on the performance of both controls and RH patients for logical inferences and total scores. For pragmatic inferences, there was a difference between control and RH patient groups (both low and high educated) and between low and high educated RH patients; there was no difference between low and high educated controls (Table 2).

**Table 2:** Comparison of performance of RH-damaged patients and controls in comprehension of inferences according to schooling level.

Type of inference	LEC			HEC			LERH			HERH		p-value	Multiple comparisons
	Mean (SD)	Range		Mean (SD)	Range		Mean (SD)	Range		Mean (SD)	Range		
Logical	4 (1.4)	1-6		5.1 (0.8)	3-6		1.3 (1.6)	0-7		2.6 (1.4)	0-5	< 0.0001	LEC ≠ HEC p=0.003 LEC ≠ LERH p<0.0001 LEC ≠ HERH p=0.001 HEC ≠ LERH p<0.0001 HEC ≠ HERH p<0.0001 LERH ≠ HERH p=0.009
Pragmatic	5.3 (1.3)	2-7		6 (0.9)	4-8		2.1 (1.5)	0-6		4.2 (1.9)	0-7	< 0.0001	LEC ≠ HEC p=0.172 LEC ≠ LERH p<0.0001 LEC ≠ HERH p=0.059 HEC ≠ LERH p<0.0001 HEC ≠ HERH p<0.0001 LERH ≠ HERH p<0.0001
Total	13.3 (3,8)	4-13		11.1 (1.5)	7-13		3.4 (2.8)	0-13		6.8 (3.1)	0-12	< 0.0001	LEC ≠ HEC p=0.010 LEC ≠ LERH p<0.0001 LEC ≠ HERH p=0.003 HEC ≠ LERH p<0.0001 HEC ≠ HERH p<0.0001 LERH ≠ HERH p<0.0001

D: standard deviation; LEC: low educated controls; HEC: high educated controls; LERH: low educated right hemisphere damage; HERH: high educated right hemisphere damage.

## DISCUSSION

The main purpose of our study was to verify the effect of schooling on the process of comprehension of inferences derived from pictorial stimulus in a sample of RH-damaged patients and normal subjects. We noted that higher levels of formal education and the absence of RH damage were associated with better scores in the comprehension of pictorial inferences in our sample. This was true for intragroup (controls high × low schooling; RH-patients high × low schooling) and intergroup (high educated controls × RH-patients; low educated controls × RH-patients) comparisons. The only exception was performance on pragmatic inferences, where no differences were found between high educated and low educated controls. Age had no effect on performance in the inferential tasks.

RH-damaged adults have been identified as having little difficulty understanding discourse when the meaning is evident (i.e., for which cognitive demands are minimal), when the inferential processing is direct, or even when the integration can proceed without revision of meaning. However, when comprehension entails consideration of extra-textual or contextual cues, which in turn require integration of information and lead to multiple and competing interpretations, the performance of this group is unsatisfactory.^27^

Educational level is a highly recognized factor impacting cognitive performance, especially as measured by neuropsychological tests, which rely to a large extent on meta-cognitive processes highly dependent on formal training (as provided by schooling).^13,15,16^

According to Warren et al.,^28^ the understanding of a particular topic is handled differently by individuals with more and less education, respectively. The greater knowledge of more educated individuals stimulates more inferences that are automatically extracted. Readers and listeners with a high mastery of the subject may pay more attention to the details of the stimulus than people with low mastery of the subject.

The relationship between schooling and comprehension of inferences was evidenced by three studies found in our literature review.^11,12,15^ In all the cited studies, schooling affected the ability to comprehend inferences.

In Myers and Brookshire's study,^11^ both normal and RH- damaged, highly educated subjects generated more appropriate inferences than individuals with average schooling while this latter group performed better than subjects with low education.

In the study of Hamel and Joanette (2007),^15^ which evaluated normal subjects and patients with RH damage, schooling influenced the subjects' performance for the comprehension of the two types of inferences: logical and pragmatic. Ribeiro et al.,^16^ who evaluated individuals without cognitive impairment, subdivided into three levels of education, demonstrated that the comprehension of visual inferences improves in proportion to the years of schooling.

According to Rosselli and Ardila,^29^ the number of years of schooling has been identified as crucial in the performance of tasks assessing memory, attention, language and executive functions. The impact of schooling has been observed even on non-verbal cognitive tasks.

In addition, the results of Meguro et al.^30^ indicated that during the aging process, schooling was more significant than age for differentiating the performance of different age groups. The authors claimed that schooling differences have consequences on brain structure, with high education producing an increased number of synapses or brain vasculature. It is notable then that increased education may be associated with significant changes in brain connections which should be investigated further.

According to Castro-Caldas et al.,^31^ an example of structural change associated with literacy is the difference in size of the corpus callosum: subjects with no education have a smaller corpus callosum, which implies less traffic information between the two cerebral hemispheres. Among the functional changes observed in more educated individuals, Reis and Castro-Caldas^32^ described that linguistic processing tended to occur in parallel in individuals with a high educational level, while more sequential processing was observed in illiterate subjects.

Parente et al.^33^ added that education increases the level of knowledge acquired by individuals, promoting greater maturity of brain structures and thus enhancing inferential abilities. Furthermore, it is known that level of education is mainly defined by number of years of study to which an individual is subjected, excluding years of school failure. However, education goes beyond quantification of years of exposure to formal education, involving reading and writing habits, personal interests, lifestyle, occupation, etc., factors that can also influence the performance of individuals on cognitive tests, specifically on the tasks proposed by this study.^33^

Our findings disclose that the inferential abilities based on pictorial stimuli are directly affected by schooling level, both in normal and brain-damaged subjects. These data should be taken into account in the devising and/or selection of visual instruments for evaluation purposes in cognitive tests using visual material, and in the selection of suitable material for possible rehabilitation of inferential processing.
